# Supplementary material for: Direct atomic-scale investigation of the coarsening mechanisms of exsolved catalytic Ni nanoparticles
Source: Nat Commun. 2025 Jul 24;16:6830. doi: 10.1038/s41467-025-61971-z (PMC12290124; doi:10.1038/s41467-025-61971-z)
Supplement: Supplementary file 1 — Supplementary Information [file 41467_2025_61971_MOESM1_ESM.pdf]

Supplementary Information

Direct Atomic-Scale Investigation of the Coarsening Mechanisms of Exsolved Catalytic Ni Nanoparticles

Dylan Jennings[1,2,3,^,*], Moritz L. Weber[4,+,#*], Ansgar Meise[2], Tobias Binninger[5], Conor Price[5], Moritz Kindelmann[1,2,6,⊥], Ivar Reimanis[7], Hiroaki Matsumoto[8], Pengfei Cao[2], Regina Dittman[4], Piotr Kowalski[5], Marc Heggen[2], Olivier Guillon[1], Joachim Mayer[2,6], Felix Gunkel[4*], and Wolfgang Rheinheimer[3*]

[1]Forschungszentrum Jülich GmbH, Institute of Energy Materials and Devices, Materials Synthesis and Processing (IMD-2); Jülich, 52425, Germany

[2]Forschungszentrum Jülich GmbH, Ernst Ruska-Centre for Microscopy and Spectroscopy with Electrons (ER-C), Jülich, 52425, Germany

[3]Institute for Manufacturing Technologies of Ceramic Components and Composites, University of Stuttgart; Stuttgart, 70569, Germany

[4]Forschungszentrum Jülich GmbH, Peter Grünberg Institute, Electronic Materials (PGI-7); Jülich, 52425, Germany

[5]Forschungszentrum Jülich GmbH, Institute of Energy Technologies, Theory and Computation of Energy Materials (IET-3), 52425 Jülich, Germany

[6]Central Facility for Electron Microscopy (GFE), RWTH Aachen University, 52064 Aachen, Germany

[7]Department of Metallurgical and Materials Engineering, Colorado School of Mines, Golden, CO 80401, USA

[8]Hitachi High-Tech Corporation, Core Technology & Solution Business Group, Ibaraki Japan

^Present address: Advanced Transmission Electron Microscopy, Faculty of Physics and Astronomy, Ruhr-University-Bochum, 44801 Bochum, Germany

+Present address: Kyushu University, Next-Generation Fuel Cell Research Center, 744 Motooka, Nishi-ku, Fukuoka, 819-0395, Japan.

#Present address: Massachusetts Institute of Technology, Department of Materials Science and Engineering, Cambridge, Massachusetts 02139, United States

⊥Present address: DTU Energy, Technical University of Denmark, Fysikvej, 2800 Kgs. Lyngby, Denmark

Corresponding authors:

Dylan Jennings (dylan.jennings@ruhr-uni-bochum.de)

Moritz L. Weber (weber.lukas.moritz.320@m.kyushu-u.ac.jp)

Felix Gunkel (f.gunkel@fz-juelich.de)

Wolfgang Rheinheimer (wolfgang.rheinheimer@ifkb.uni-stuttgart.de)

Ex-Situ Thin Film Characterization

Epitaxial STNi thin films are deposited by RHEED-PLD (reflection high-energy electron diffraction-controlled pulsed laser deposition). A layer-by-layer growth mode becomes apparent by *in-situ* monitoring of the (0 0) specular spot (Supplementary Fig. 1a). After the deposition, a predominant 2D RHEED diffraction pattern remains visible, while the emergence of a less distinct, superimposed 3D diffraction pattern indicates a slight increase in surface roughness after the deposition of 150 nm of STNi (Supplementary Fig. 1b). This observation is consistent with investigations of the surface morphology by atomic force microscopy (Supplementary Fig. 1c). While a stepped surface morphology remains visible (distinct surface step is visible in center of the image), an average surface roughness of RMS = 842 ± 108 pm was detected. High resolution X-ray diffraction analysis reveals a (002) thin film peak at slightly lower diffraction angles as compared to the (002) diffraction peak obtained from the single crystalline Nb:STO substrate (Supplementary Fig. 1d). Hence, minor compressive strain is induced by epitaxial growth, and as a consequence of a slightly larger lattice parameter of STNi relative to the substrate. Moreover, distinct Laue oscillations are visible in the vicinity of the (002) diffraction peaks indicating a high crystallinity of the STNi thin films and high coherence of the Nb:STO-STNi and STNi-air interfaces.

Supplementary Figs. 2, 6-9 detail additional pre-characterization of the STNi thin films. In Supplementary Figs. 2 and 6, EDS mapping of the thin film shows Ni inhomogeneities which are present in the film. The plan-view EDS mapping (Supplementary Fig. 4) demonstrates a web-like structure of increased Ni, with NiO_x secondary phases present at the triple-junctions of the web-pattern. When observing the cross-section EDS scan (Supplementary Fig. 2), it can be seen that the Ni inhomogeneities run through the full thin film. No significant changes in Sr, Ti, or O chemistry can be seen from either orientation (at this magnification).

A variety of high-resolution (HR) analyses have also been done on the as-grown STNi film to characterize the embedded NiO_x nanocolumns. Supplementary Fig. 5 details several additional high-resolution HAADF images of embedded nanocolumns. The columns all have similar dimensions, with a width of around 2 nm, but show a variety of morphologies. The embedded nanostructures are observed to impact the strain state of the film along with the chemical bonding environment

(Supplementary Fig. 8). Real-space column-ellipticity mapping of an HR HAADF image (Supplementary Fig. 8b) shows strain in the {110} directions in the strontium titanate lattice within ≈5-10 lattice planes of the nanostructure. Electron energy loss spectroscopy (EELS) mapping was performed on the region of the HAADF image shown in Supplementary Fig. 8c. When comparing EELS spectra of the O K edge between the Ni:$SrTiO_3$ region and the Ni-rich region (Supplementary Fig. 8d), there is a distinct change in the fine structure of the edge, indicating that the oxygen bonding environment is altered. When mapping the spacing between the two peaks in the O K fine structure (Supplementary Fig. 8(e)) over the full image, it is clear that peak shifts occur even in the brighter regions which don't contain any obvious nanostructure. The change in the O K fine structure in these locations is likely indicative of increased oxygen vacancy concentration in regions of increased Ni content [1]. In an attempt to image the influence of the NiO_x nanostructures on the oxygen sublattice in the Ni-doped strontium titanate film, integrated differential phase contrast (iDPC) imaging was done on the as-synthesized thin films (Supplementary Fig. 9). The oxygen sublattice is clearly fully coherent up until the edge of the NiO_x nanostructure, and there is no noticeable influence from the nanostructure on the sublattice. Supplementary Fig. 3 shows an SE and HAADF image of a NiO_x nanostructure taken during an in-situ experiment in air at 500 °C, showing that the nanostructure visible in the HAADF image is completely buried within the $SrTiO_3$ thin film.

Thermodynamic modelling of Ni solubility in $SrTiO_3$

A thermodynamic model of Ni-$SrTiO_3$ solid solution formation was applied to understand the formation of NiO_x nanostructures in the as-grown thin film. Three separate models were compared to compute the Margules interaction parameter *W*. The models are as follows: model 1: consideration of ionic sizes of Ti^{4+} (0.605 A) and Ni^{4+} (0.48 A) ions[2,3], model 2: considering volumes of endmembers[4] and model 3: direct DFT computation. Similar models have been successfully applied previously to understand solubility limits in various ceramic and Ni-based materials[5,6]. To validate the applied computational setup, the formation enthalpy of $SrTiO_3$ (STO) was computed, and is consistent with experimental values[7]. In addition, the computed formation enthlapy is reported for $SrNiO_3$ in the cubic and the hexagonal phase most stable in the $BaNiO_3$ system[7,8], as well as $SrNiO_{2.5}$ as shown to form

experimentally in previous studies[7]. The formation enthalpy for Sr-Ni-O (SNO) compounds has not been measured experimentally. The results are reported in Supplementary Table 1.

Supplementary Table 1: The computed formation enthalpy H^f of STO and SNO from oxides (SrO, TiO_2, NiO) estimated from the DFT total energies. The experimental value for STO comes from Zinkevich[7]. The last column reports estimates of formation Gibbs free energy G^f, made by considering the entropy of molecular oxygen gas[9], experimental oxygen partial pressure of 0.108 mbar, and a temperature of 650 °C. The values are reported in kJ mol^{-1}.

Compound	Calculated H^f	Experimental H^f	Estimated G^f
$SrTiO_3$	-127	-132+/-9 [7]	-127
$SrNiO_3$ (hexagonal)	-207		-19
$SrNiO_3$ (cubic)	-134		+54
$SrNiO_{2.5}$	+10		+198

To assess the possibility of solid solution formation, the parameter W was estimated by applying the strain energy based-model of Mogilevsky as well as Kowalski and Li[2,4]. As the most stable phase of SNO (hexagonal) has a different structure compare to the most stable phase of STO (cubic), the W parameter is computed as:

$$W=W_0+\Delta E_{c\text{-}h} \quad \textbf{Supplementary Eq. 1}$$

where W_0 is the Margules interaction parameter obtained assuming both solid solution endmember phases as cubic and $\Delta E_{c\text{-}h}$ is the energy difference between the cubic and hexagonal phases of SNO. The computed value of $\Delta E_{c\text{-}h}$ is 76.7 kJ mol^{-1}. The results are provided in Supplementary Table 2, together with the solubility limit computed from the derived W parameters by applying the solid solution model of Mogilevsky[2]. The two scenarios of doping on the Ti and Sr sites were considered. Our calculations, as anticipated, show that doping on the Ti site is preferred by 349 kJ mol^{-1} compared to doping on the Sr site. The obtained values of the W parameter are large and hence the derived solubility limits indicate small amounts of incorporation of Ni and ultimately a phase separation. We also note that because the formation of oxygen defects in highly endothermic, we consider direct cation exchange as the major solid solution formation process. The W_0 parameters derived directly from DFT are large and negative, resulting from the lowered favorability of the cubic $SrNiO_3$ phase. The very small solubility limit of Ni in STO supports a phase separation into the STO and SNO

phases. It is noted that the computed formation enthalpy of $SrNiO_3$ is negative and large, and therefore would indicate the formation of the NiO_x phase, as is seen in the experimental STEM images (Fig. 1).

Supplementary Table 2: Calculated values of W_0 for doping STO with Ni. The solubility limit has been computed assuming T=650 °C. The Young's modulus of 282.9 GPa used in Model 1 and Model 2 has been derived here with the Voight-Reuss-Hill approximation following procedure described in Ji et al. [10].

Model type	W_0 (kJ mol^{-1})	W (kJ mol^{-1})	Solubility limit
Ionic Sizes (model 1)	8.8	87.4	1.2 10^{-9}
Endmember Volumes (model 2)	9.0	85.7	1.2 10^{-9}
DFT (model 3)	-31.1	45.6	2.1 10^{-7}

To simulate the 'nanocolumn-associated' Ni particles, the same model Ni particle was attached to a Ni column pedestal. The respective adhesion energy of the Ni particle to the Ni column was calculated as 0.42 eV $Å^{-2}$ (2.40 eV per interfacial Ni atom, or 86.5 eV total), about three times stronger than the adhesion of the Ni particle to the pristine $SrTiO_3$ surface. The drastically different adhesion energies can explain the static pinning of nanocolumn-associated Ni particles in contrast to the high mobility of 'pristine' Ni particles, as observed in experiment.

Supplemental in-situ Characterization

Several additional figures are presented to support the in-situ findings of the manuscript. During heating, imaging was done at the edge of the sample to ensure that the sample surface was fully crystalline (Supplementary Fig. 15). The exsolution and particle growth kinetics are shown in Supplementary Fig. 11, which compare qualitatively well with the literature for this system[11]. In particular, the sample shows very fast exsolution kinetics, resulting in an initially high particle density of small sized nanoparticles (Supplementary Fig. 11c,d), followed by coarsening which reduces the particle density significantly. Several exemplary nanoparticles after exsolution are displayed in Supplementary Fig. 12. While some of the particles are too small to observe lattice structure in the SE images, all of the particle shapes are consistent with a cube-on-cube epitaxial relationship between the particles and the strontium titanate support.

In certain areas, clear surface steps in the strontium titanate surface were visible during SE imaging (Supplementary Fig. 19), likely a result of slightly misangled cuts made in the FIB, resulting in differences in step widths between SE imaging and AFM imaging in Supplementary Fig. 1. Some particles are attached to step-edges, while others are present on the atomically-flat planes between the

surface steps. Under an assumption that the surface roughness in Supplementary Fig. 19 is limited to surface steps of 0.39 nm in height which are noted by the red lines in Supplementary Fig. 19(c), the RMS roughness is calculated to be ≈0.4 nm, which is on the same order as the roughness of the as-grown thin film. The roughness observed during SE imaging suggests that the surface character during in-situ measurements is very comparable (from the perspective of roughness) to the native film surface. The strontium titanate surface facets have been observed to rearrange during the in-situ experiments (refer to Supplementary Fig. 20). Alternating the atmosphere between vacuum and hydrogen was shown to have a slight impact on the faceting behavior of the Ni particles (Supplementary Fig. 21). Upon the introduction of hydrogen, the roundness of the particles is observed to increase, similar to what has been seen ex-situ in similar samples [12]. As has been shown in other systems [13], it is anticipated that the decrease in pO_2 results in a reduction of the anisotropy in the surface energy of the metallic Ni particles.

Supplementary Fig. 22 details EDS mapping which was done at 400 °C from a pristine particle after exsolution. No significant other elemental signals were observed which matched with the particle location in the map. In addition to EDS mapping, a control liftout was prepared by the same method from a Nb:$SrTiO_3$ single crystal. After going through the exact heating experiment (the control sample and the STNi sample liftout were placed on the same MEMS chip), no particles are visible on the surface of the control lamella (Supplementary Fig. 23). This result confirms that no particles are appearing as an artefact resulting from FIB-preparation.

Evaluation of Beam Effects during in-situ Experiments

Understanding the effect of the electron beam on material behavior during in-situ STEM experiments is critical, however no significant beam effect was observed during the experiments presented in this work. For oxide species the presence of the electron beam is known to induce local reduction[14]; a local reduction could result in enhanced exsolution around regions which are exposed to the beam, but no evidence of the beam impacting the exsolution process was observed in the current experiment. Several pieces of evidence are presented here to demonstrate that beam effects do not significantly influence the observations in this work.

Firstly, the dynamic processes observed (particle migration and Ostwald Ripening) are qualitatively and quantitatively consistent at varying electron dose rates. Particle migration was observed at relatively low dose (350 e^{-} $Å^{-2}$ s^{-1}), Supplementary Movie 6), intermediate dose rates (5.0×10^{3} e^{-} $Å^{-2}$ s^{-1}, Supplementary Movies 1 and 3), and high dose rates (8.9×10^{4} e^{-} $Å^{-2}$ s^{-1}, Supplementary Movies 4 and 5). Quantification of particle migration at both low and intermediate dose rates shows similar kinetic behavior – that is, behavior which is consistent with a random-walk model. Refer to Supplementary Fig. 25 for a comparison which shows Brownian motion type kinetics at the two different dose rates. Ostwald ripening processes were also observed at low, intermediate, and high dose rates.

In addition to recording consistent behavior at different dose rates, features similar to those observed during live imaging were also seen in regions of the sample which were not continuously exposed to the electron beam. Supplementary Fig. 14 shows contrast features surrounding non-live-imaged particles which are qualitatively comparable to contrast features observed in Fig. 5d,e, a region which was continuously imaged. Additionally, Figure 5a-c shows a before and after of a region which was not continuously imaged, with clear evidence of the Ostwald Ripening processes that are seen in live imaging (particle redissolution and particle growth).

Finally, the observations made while live imaging the samples are consistent with physical theories for nanoparticle growth kinetics, and fit to observations from ex-situ work on the same material system. Nanoparticle exsolution was first observed at 400 °C, consistent with ex-situ work. Furthermore, the temperature at which nanoparticles become mobile on the surface is consistent with expectations, at a temperature of around 500 °C (based on work from Hu and Li[15] one expects mobility to appear around 430 °C). In addition to in-situ experiments in the STNi system, comparable in-situ experiments were done in the STNNi system (a Ni and Nb co-doped strontium titanate thin film). When comparing the particle evolution between the two material systems, they behave exactly as expected. Nanoparticles in the STNNi system exsolve more slowly, but are also much more stable to coarsening. There were also no observations of particle migration in the STNNi system, supported by ex-situ work which posits that particle migration only occurs in the STNi material system. The comparison between this work and the corresponding literature is shown in Supplementary Fig. 24.

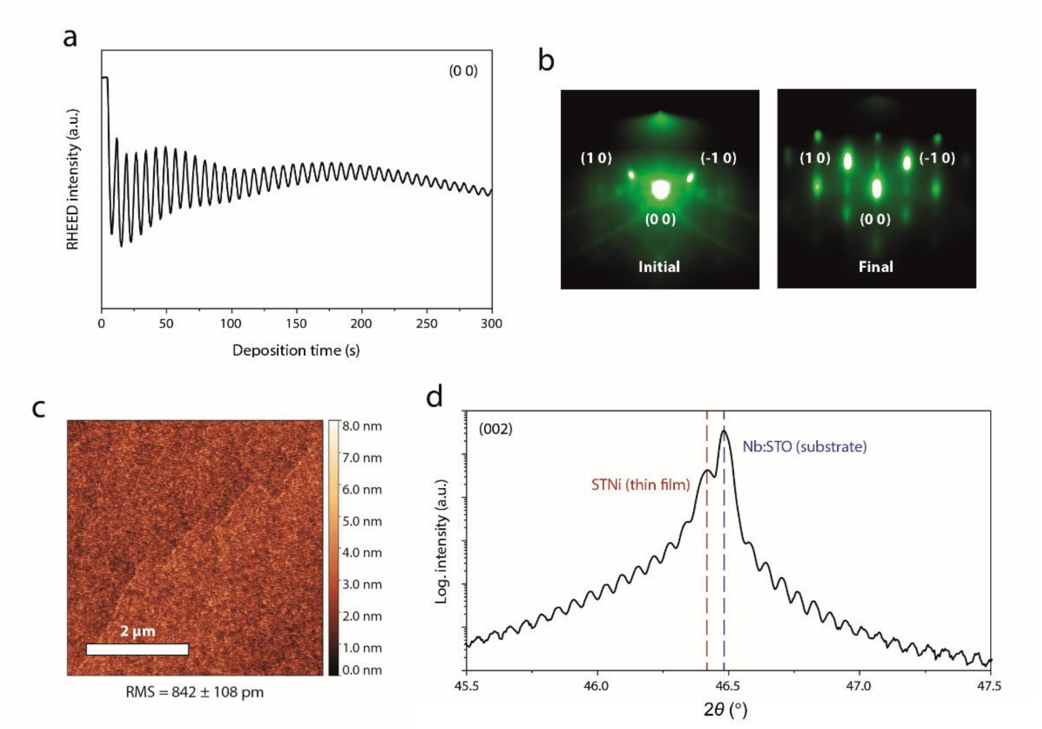

Supplementary Figure 1: Epitaxial growth of 150 nm thick STNi thin films on TiO_2-terminated Nb:STO and representative surface and crystallographic characterization. (a) RHEED intensity evolution during the initial growth of STNi as revealed by monitoring of the (0 0) specular spot of the RHEED surface pattern. (b) RHEED diffraction pattern obtained from the surface i.e. the Nb:STO substrate before the deposition (initial) and after the deposition of a STNi thin film (final). (c) Representative surface morphology of the as-deposited STNi thin film revealed by atomic force microscopy. (d) High-resolution X-ray diffraction analysis of STNi in 2Theta-omega geometry performed in the vicinity of the (002) substrate and (002) thin film diffraction peaks.

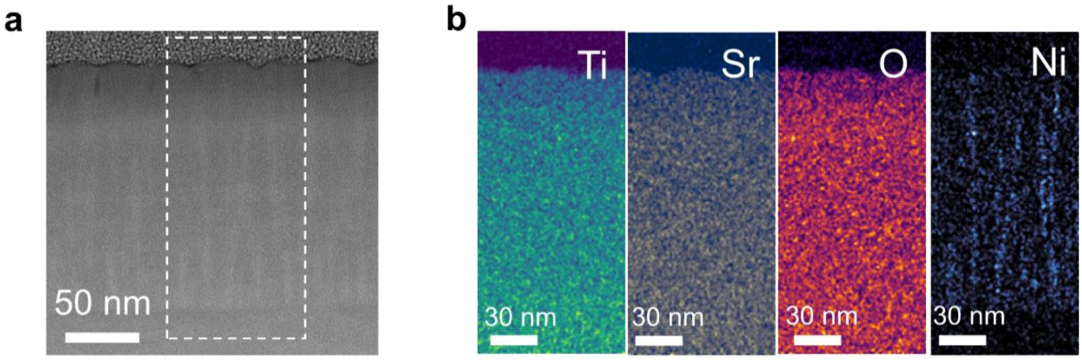

Supplementary Figure 2: HAADF image (a) and EDS mappings (b) (taken from white box) of as-deposited defect structures in STNi thin film from the cross-sectional view.

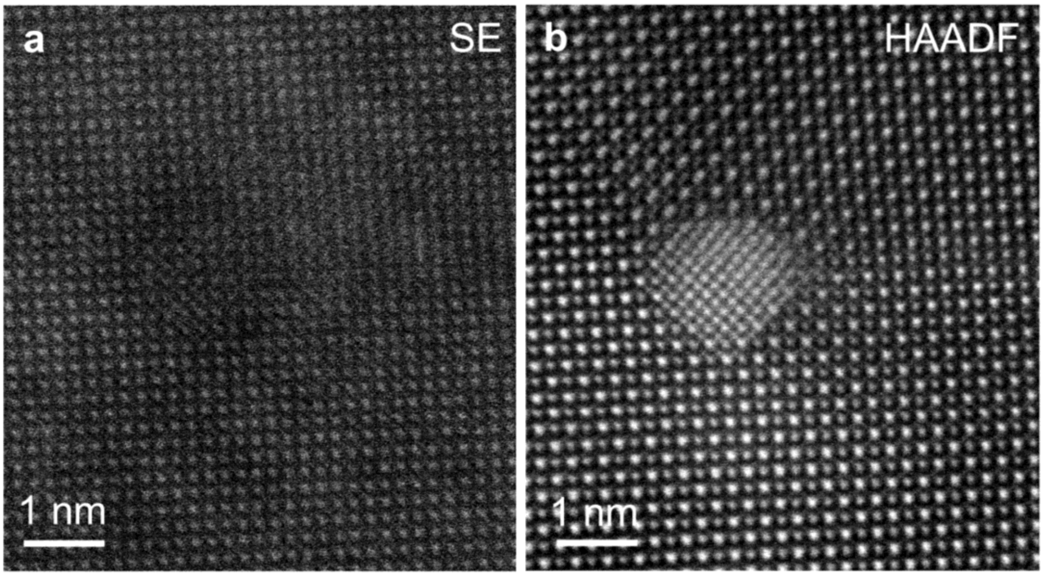

Supplementary Figure 3: (a) SE and (b) HAADF images of an embedded NiO_x nanostructure collected during in-situ heating, showing the nanostructure does not rise above the surface of the thin film. Experimental conditions during acquisition: T = 500 °C,

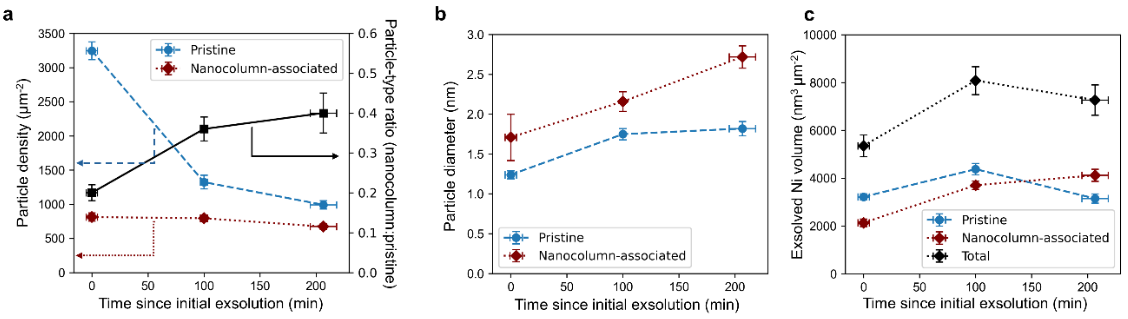

Supplementary Figure 4: Complete comparison of exsolution behavior during the in-situ experiment. (a,b) data from Fig. 2, combined with a plot of the total volume of exsolved Ni over the course of the experiment (c). The amount of exsolved Ni increases slightly over the course of the experiment. An average of 46 particles per timestep were evaluated for the data, and error bars represent the standard error of the mean of the measurement.

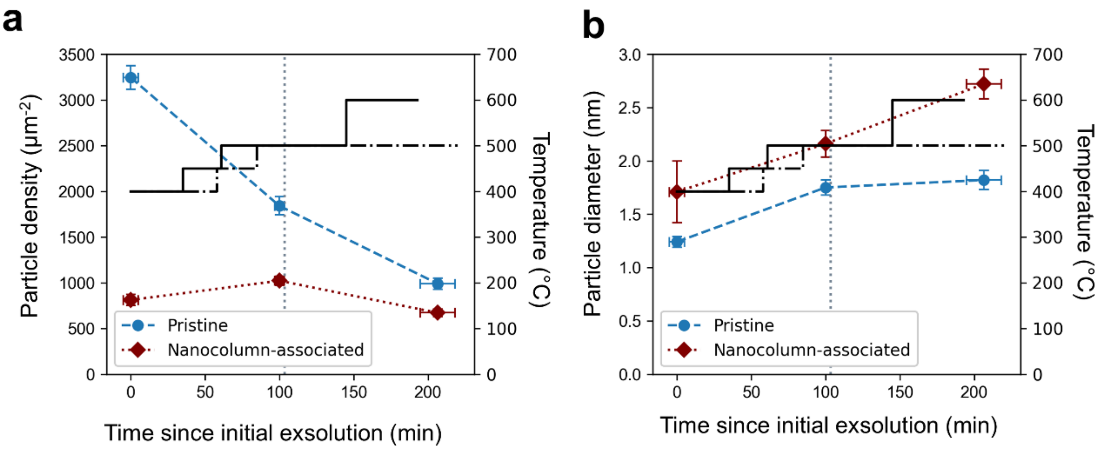

Supplementary Figure 5: Particle evolution in terms of density (a) and diameter (b) from Figure 2, with experimental temperature ramps included. The two black lines correspond to the temperature ramps of the two separate experiments from which the data was collected. The vertical line indicates the point in which the atmosphere was changed from 1 Pa H_2 to vacuum (within an error of ±6.5 min). An average of 46 particles per timestep were evaluated for the data, and error bars represent the standard error of the mean of the measurement.

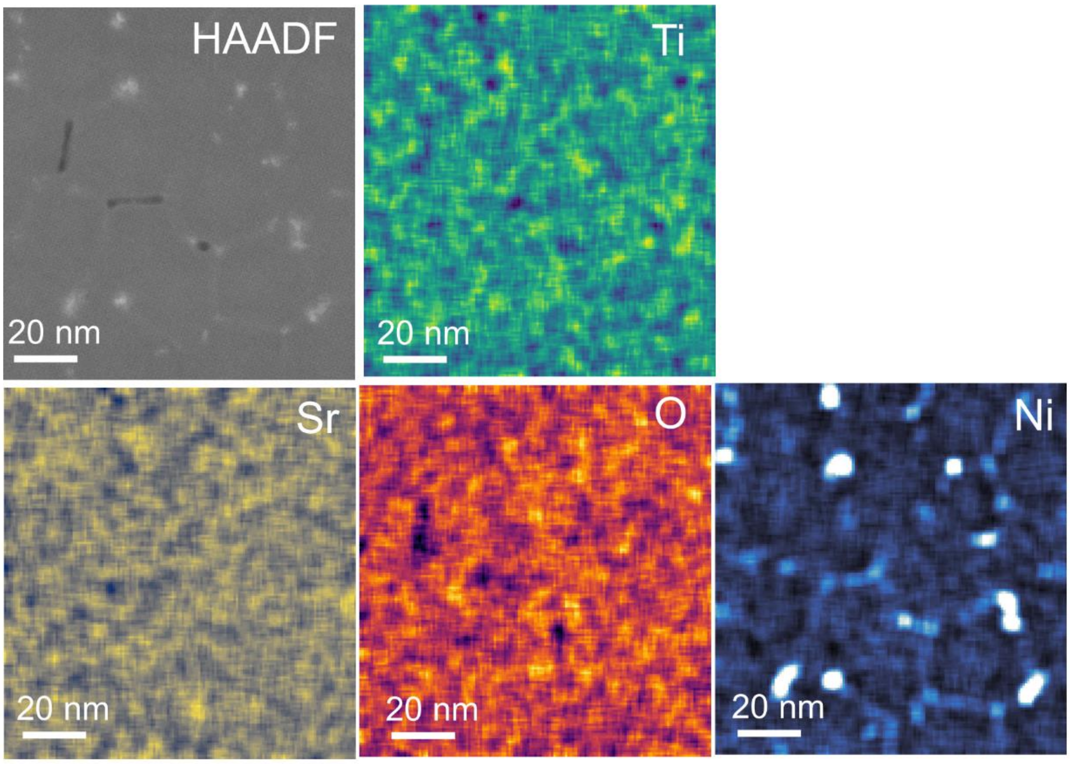

Supplementary Figure 6: Complete EDS mappings of as-deposited Ni-rich structures in the STNi thin film from the plan-view.

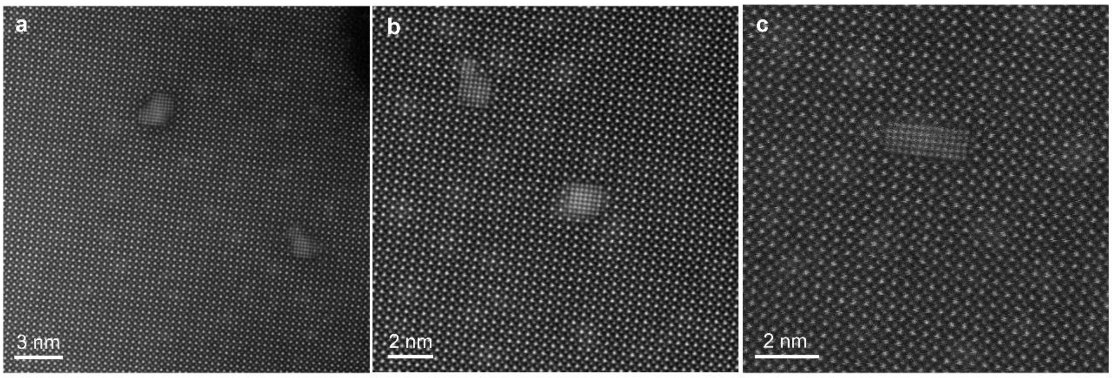

Supplementary Figure 7: Select plan-view high-resolution HAADF images of additional NiO_x nanocolumns in the as-grown STNi thin film.

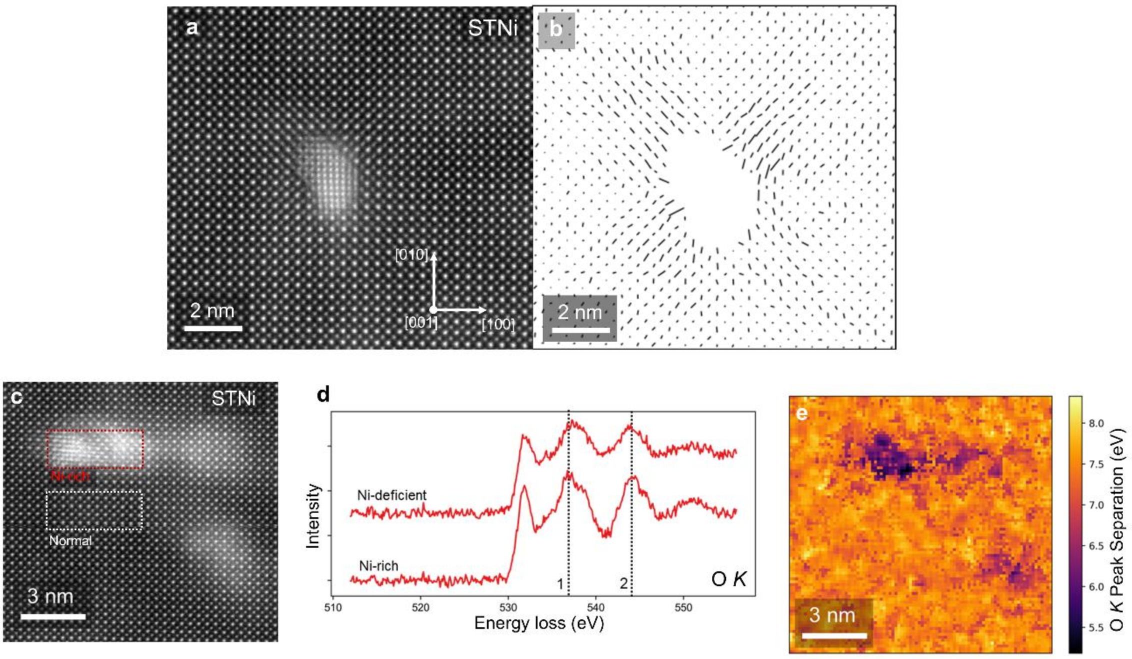

Supplementary Figure 8: High resolution STEM imaging and analysis of a plan-view liftout, detailing defect structure of the as-grown STNi film. (a) a NiO_x nanocolumn representative of those in the STNi film. (b,d-f) further analysis of the STNi sample, including (b) mapping of the ellipticity of cation columns in the region around the inclusion in (a) which shows strain at the interface. Another HRSTEM image is shown (d) with a nanocolumn and a section of higher Ni content (i.e. brighter region). (e) Oxygen *K*-edges from a Ni-rich area and a Ni-deficient area of the image in (d), and (e) a mapping of peak separation between the two O *K* peaks (notated 1 and 2 in (e)) from the full area in (d), showing that the peaks shift closer together in the Ni-rich regions of the film.

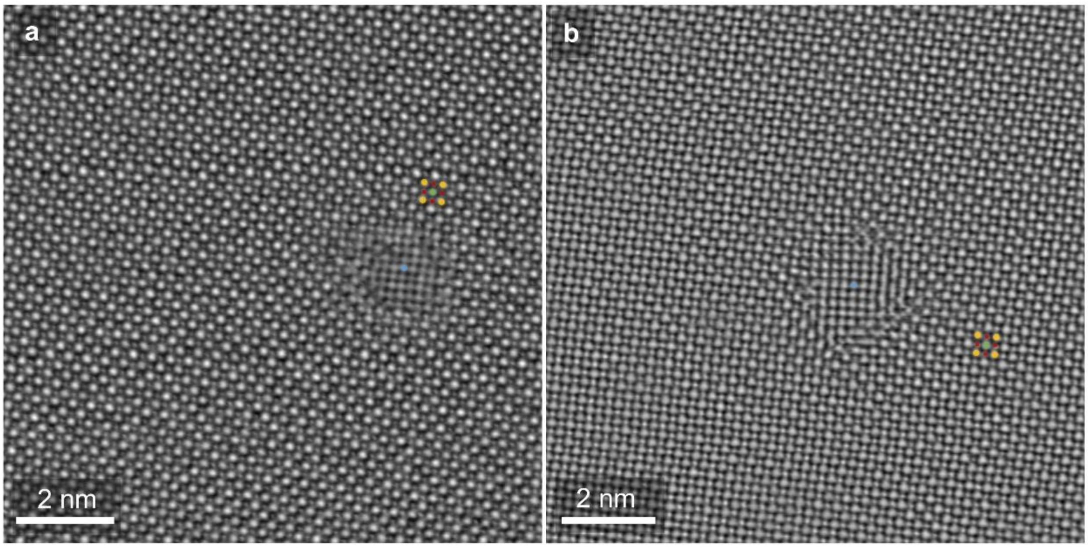

Supplementary Figure 9: iDPC STEM images of NiO_x nanocolumns in a plan-view liftout of the STNi thin films. One unit cell of strontium titanate is indicated on each figure (yellow = Sr, green = Ti/O, red = O). One Ni/O column in (a,b) is indicated with a blue

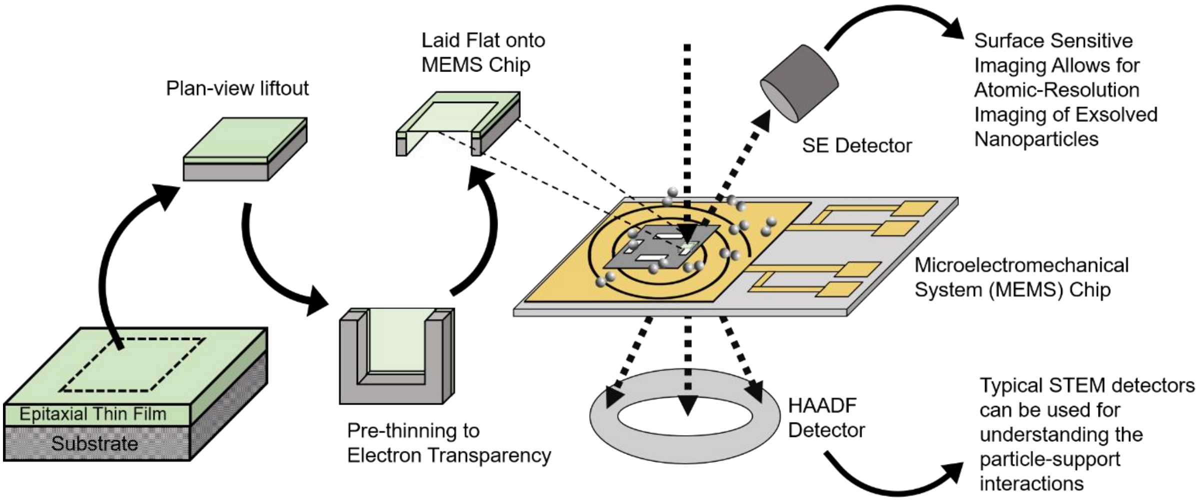

Supplementary Figure 10: Pictogram emphasizing the setup for in-situ STEM exsolution experiments. From left to right: a plan view liftout of the film is made, then attached to a MEMS heating chip (not shown). Two detectors for imaging are utilized during environmental STEM experiments: secondary electron (SE), allowing for imaging of exsolved particles on the surface, and high angular annular dark field (HAADF), giving information the structure of the substrate below the exsolved particle.

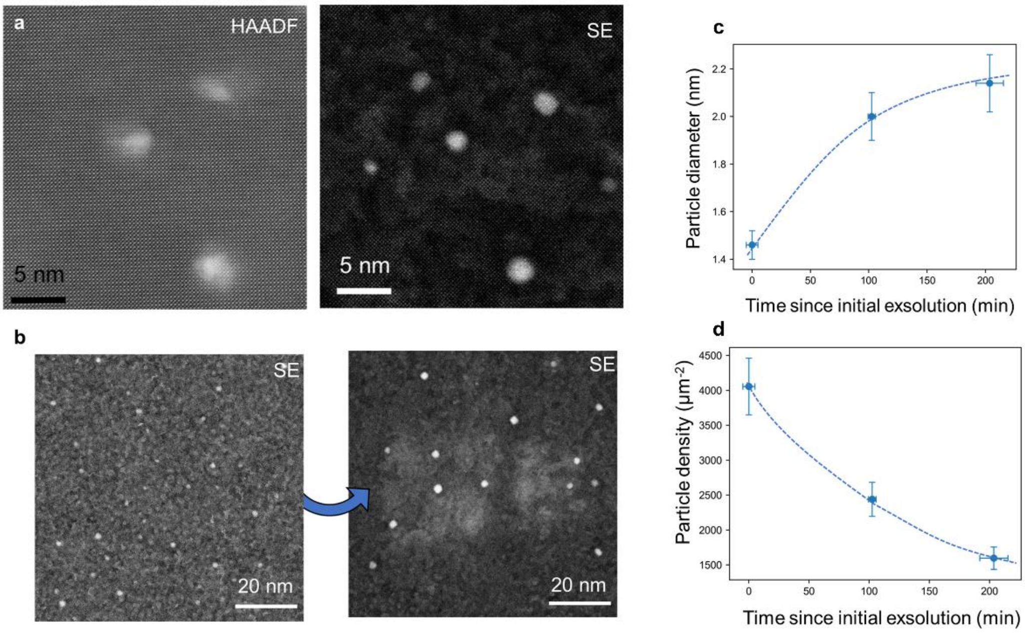

Supplementary Figure 11: in-situ STEM exsolution experiments were done on plan-view samples for the STNi sample. (a) exemplary HAADF and secondary electron (SE) images of exsolved particles in the STNi sample, and (b) two SE images showing particle coarsening during the experiment (note: while at the same scale, the images are taken from different locations on the sample). General trends for particle diameter (c) and density (d) are presented. Exsolution was first noted at 400 °C, and the maximum temperature during the experiment was 600 °C. Error bars are the standard error of the mean of the averaged values. The images in (a) were collected at 500 °C in vacuum and those in (b) were collected at 400 °C (pre) and at 500 °C (post), both in hydrogen. An average of 46 particles per timestep were evaluated for the data, and error bars represent the standard error of the mean of the measurement.

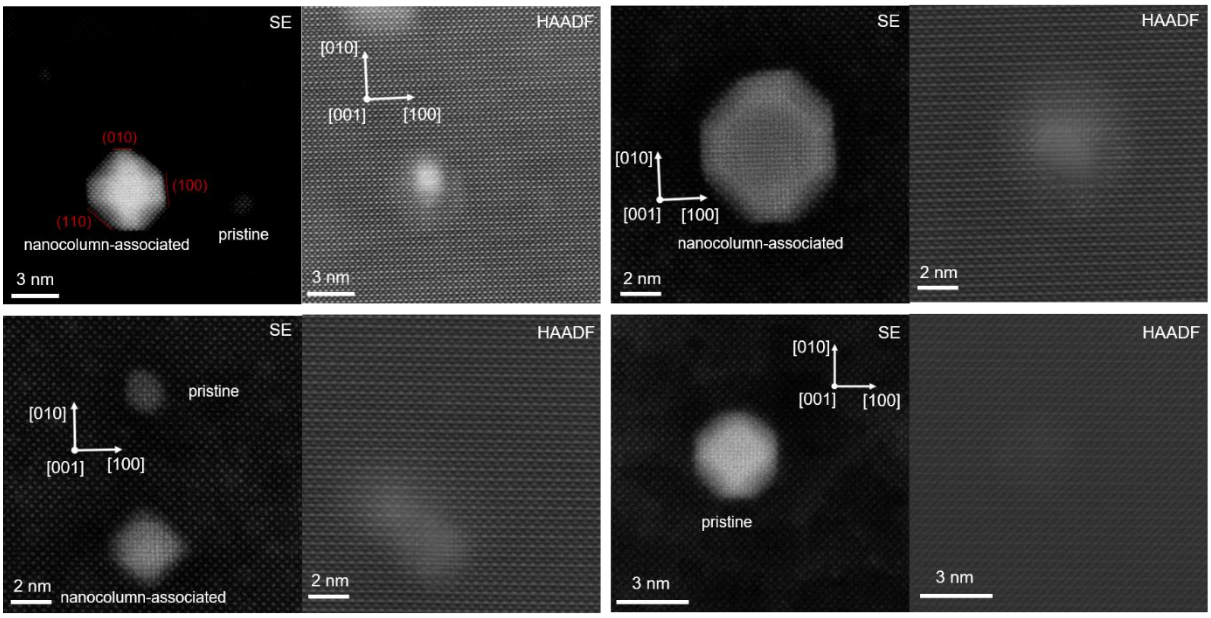

Supplementary Figure 12: Select high resolution images of nanocolumn-associated and pristine particles. All particles have similar faceting behavior, consistent with a heteroepitaxially aligned Ni nanoparticle which has exsolved. All images were collected at 500 °C in vacuum.

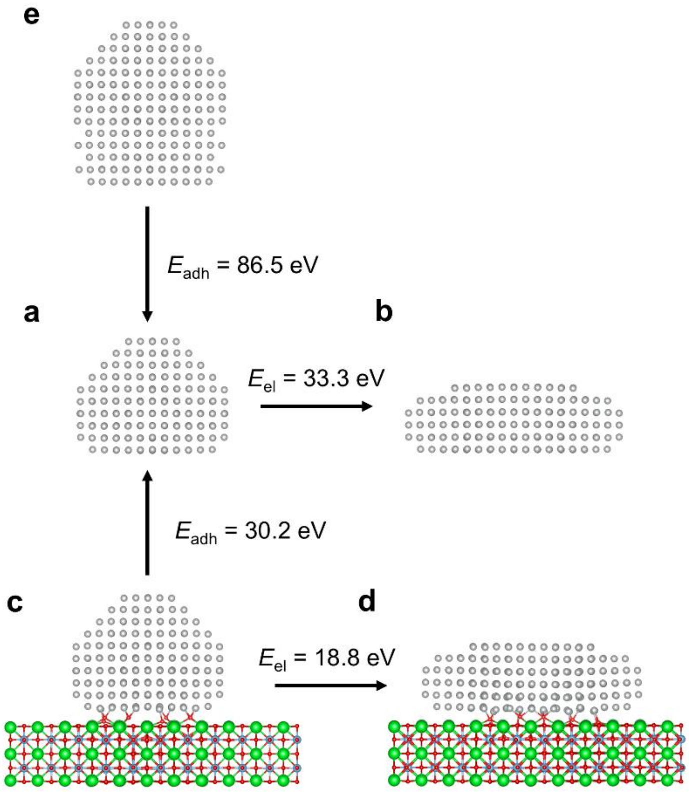

Supplementary Figure 13: Full density functional theory results, showing adhesion and elongation energies and relaxed structures: the initial nanoparticle in vacuum (a) and attached to the support (c), and the elongated nanoparticle in vacuum (b) and attached to the support (d). Additionally, (e) shows the particle attached to a Ni nanocolumn of the same interface diameter. The adhesion energy, E_{adh} is the energy required to remove the particle from the substrate, and the elongation energy, E_{el}, is the energy required to elongate the particle. Color code: green = Sr, blue = Ti, red = O, grey = Ni.

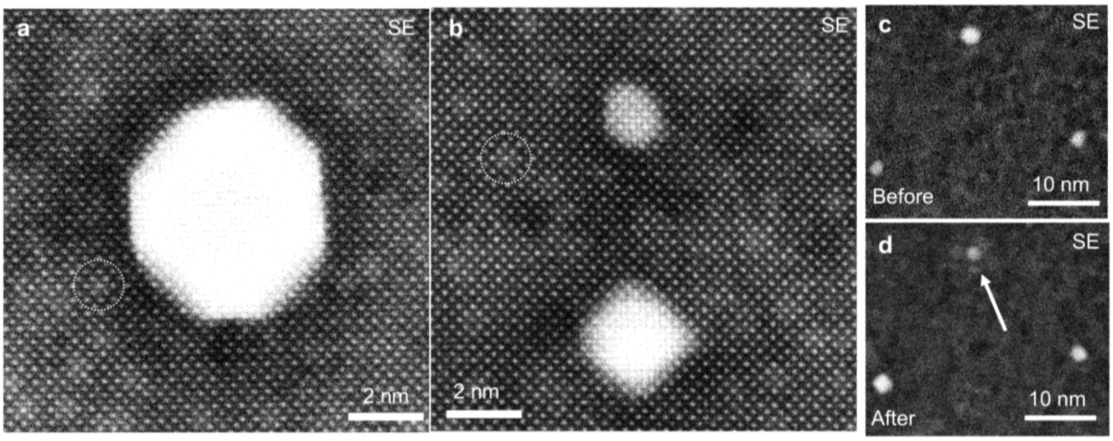

Supplementary Figure 14: Surface contrast features similar to those in Figure 4(e,f) in regions which have not been continuously exposed to the electron beam. (a,b) high resolution SE images with increased contrast showing small 'islands' of bright contrast (circled in white), hypothesized to be Ni species at or near the surface. (c,d) Before (*t* = 0, 450°C) and after (*t* = 22 min, 500 °C) SE images of a region with three particles, where one has shrunk while the other two have grown. The particle which shrunk shows a cloud of brighter contrast around it after redissolution (white arrow in (d)). Experimental conditions: (a,b) 500 °C, vacuum; (c) 450 °C, hydrogen; (d) 500 °C, hydrogen.

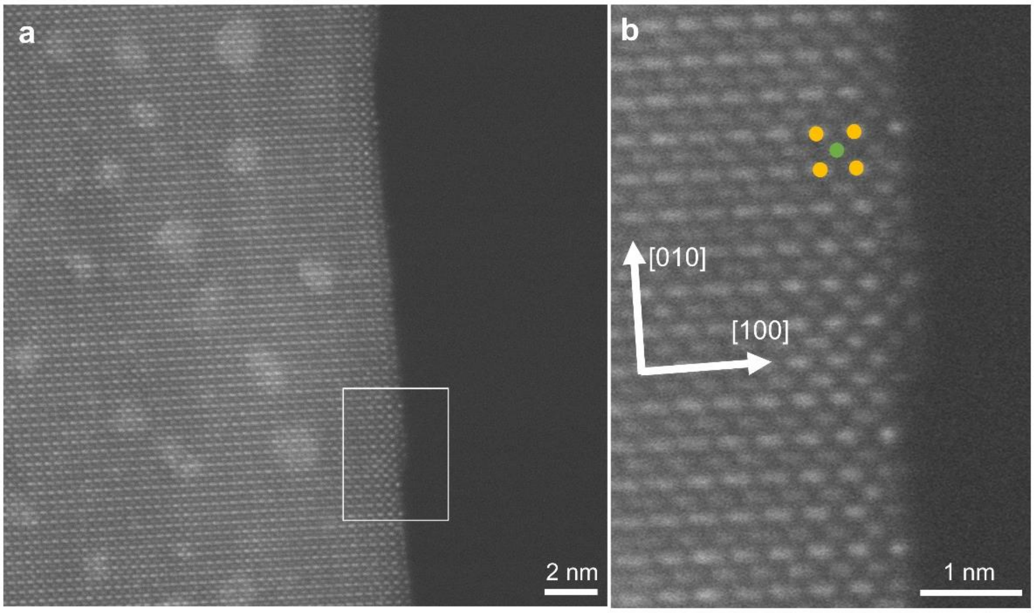

Supplementary Figure 15: (a) High angle annular dark field imaging of the sample edge at 300°C in vacuum, which shows a surface with no amorphous layer. (b) a zoom-in from the white box in (a) indicates that the {100} type surfaces are fully Sr-terminated. A single unit cell is notated in (b): yellow = Sr, green = Ti/O.

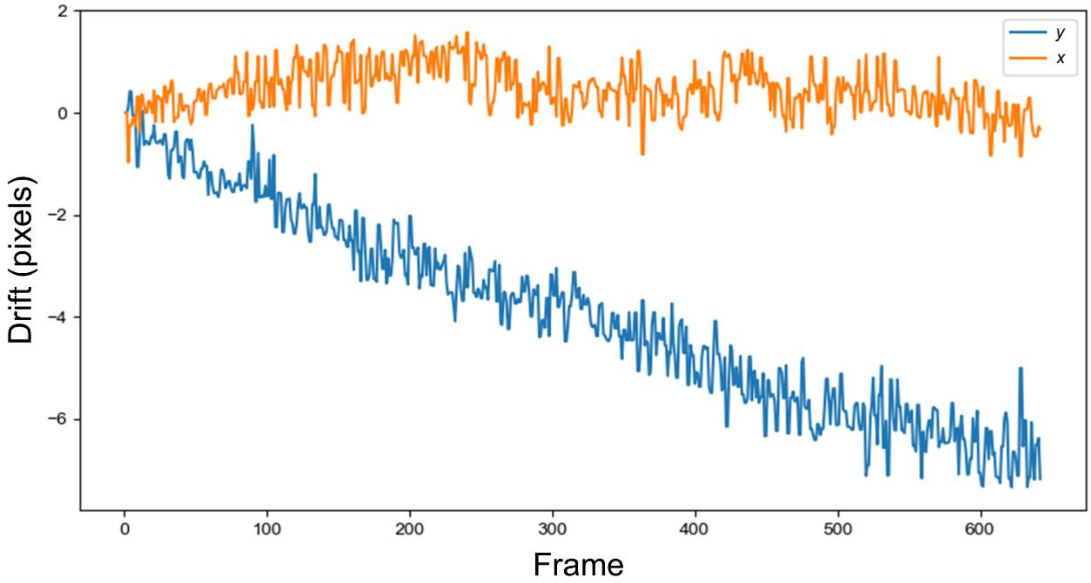

Supplementary Figure 16: Drift measurement in the *x* (orange) and *y* (blue) dimensions taken from a stable particle in Fig. 3. The *x*-drift was determined to be a result of measurement noise, and thus was used to calculate a minimum significant jump distance cutoff for kinetic analysis of the mobile particle.

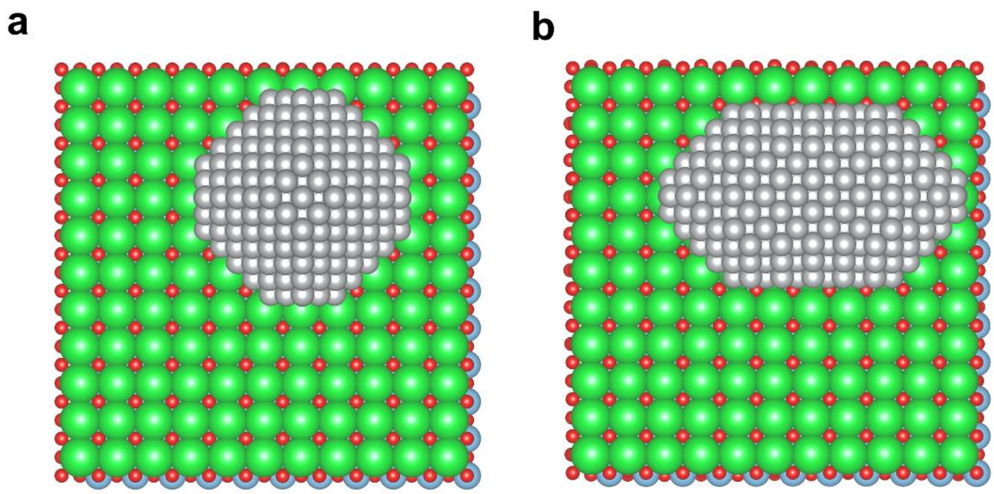

Supplementary Figure 17: Supercells built for VESTA simulations, consisting of Ni particles with 409 atoms and $SrTiO_3$ slabs with 1452 atoms. (a) the ‘initial’ particle, built with a size, a cube-on-cube orientation relationship, and faceting to replicate experimental images. (b) The ‘elongated’ particle, which was modeled to recreate the experimental observation in Fig. 4(b) while maintaining the same number of total atoms. The color code is as follows: green = Sr, blue = Ti, red = O, grey = Ni.

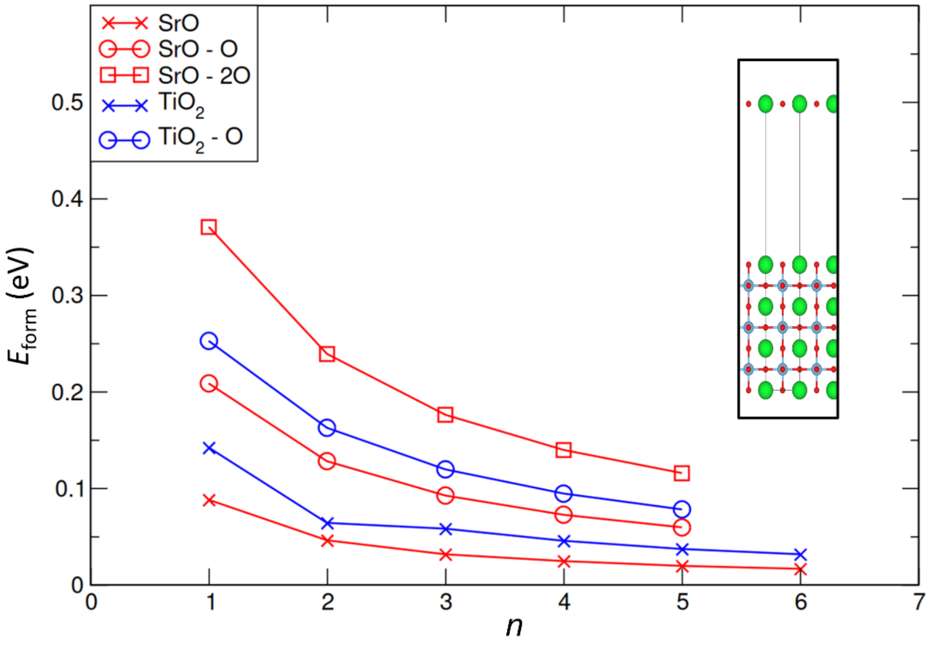

Supplementary Figure 18: DFT simulations of surface energy for a variety of terminations of a $SrTiO_3$ {100} surface in vacuum with varying thickness in terms of unit cells, n. The inset in the figure shows an example slab of n=3 with a SrO termination. Simulations indicate that the SrO surface is most favorable, without any adsorbed oxygen atoms. As a compromise between accuracy and computational time, *n*=2 was used for full DFT calculations. The color code for the inlay is as follows: green = Sr, blue = Ti, red = O, grey = Ni.

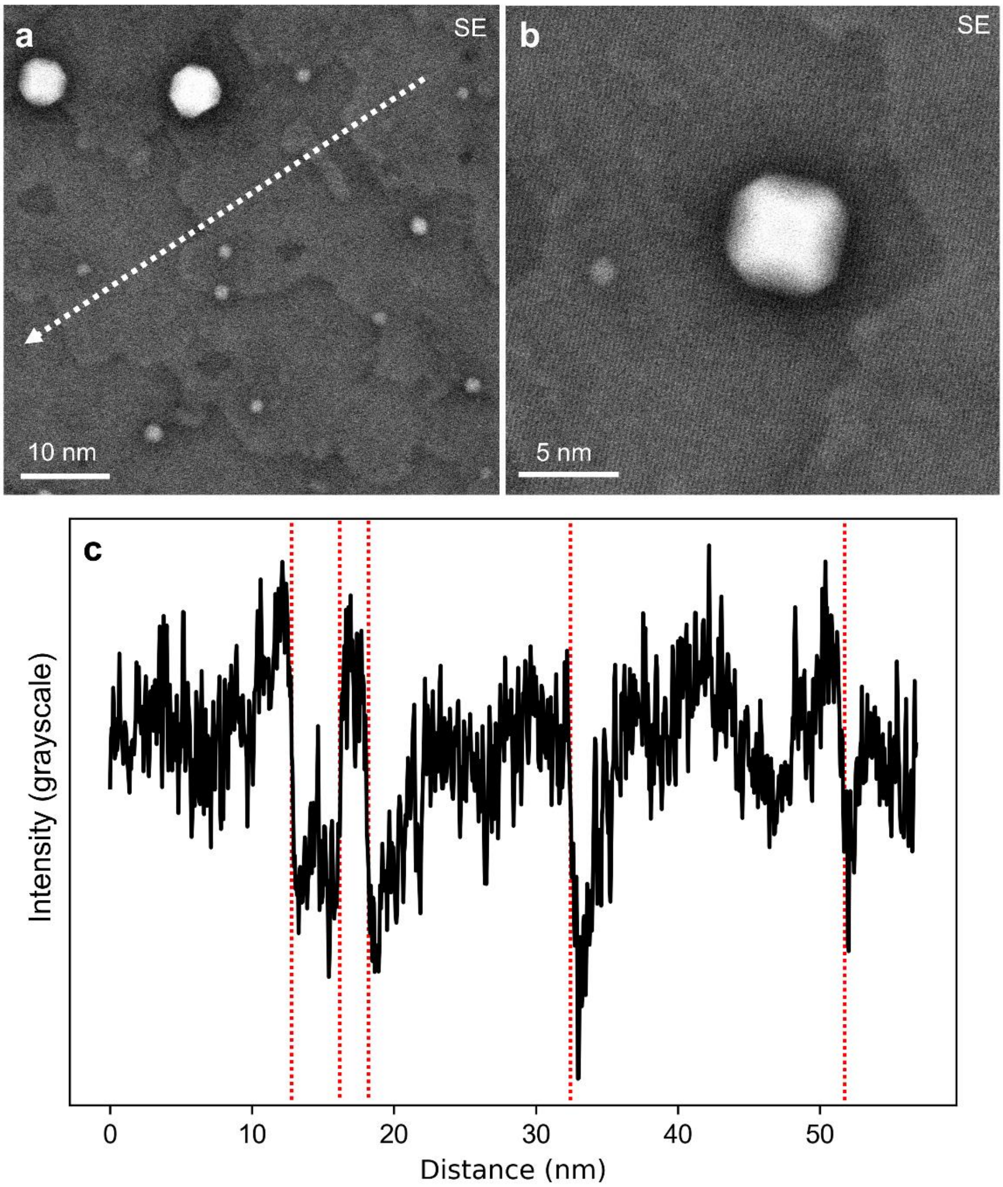

Supplementary Figure 19: Areas of the sample were present with clear surface steps. Some particles are observed to sit at surface step-edges, while others are on atomically-flat sections. The images in (a,b) were collected at 700 °C in vacuum. Lattice planes are visible in (b), but the lattice was mis-aligned due to lamella bending during heating. (c) line scan of contrast along the arrow in (a), showing several surface steps indicated by red dashed lines.

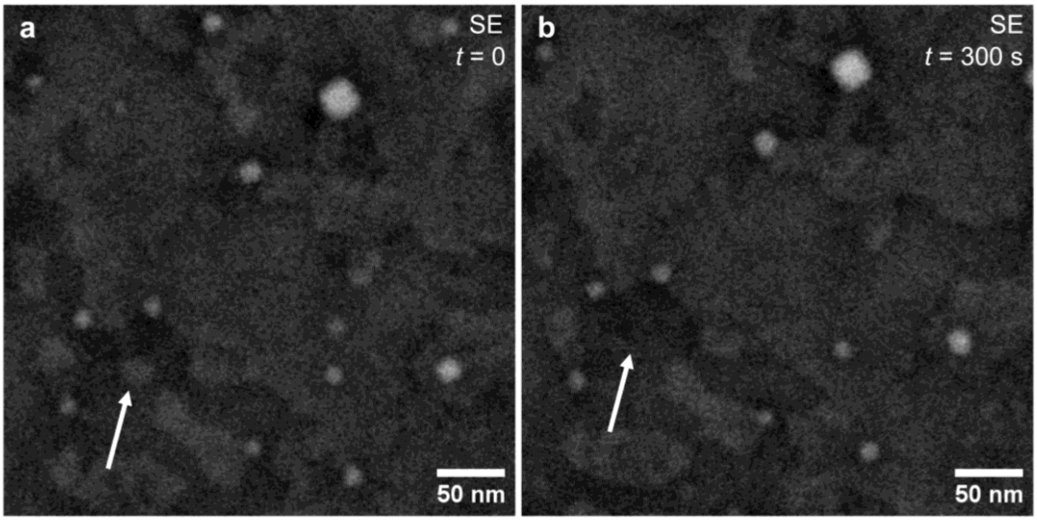

Supplementary Figure 20: Before (a) and after (b) secondary electron images showing flattening of the $SrTiO_3$ support at 700 °C in vacuum. The arrow indicates an $SrTiO_3$ surface island which disappears after 5 min. Surface step structures are also observed to evolve in other parts of the image.

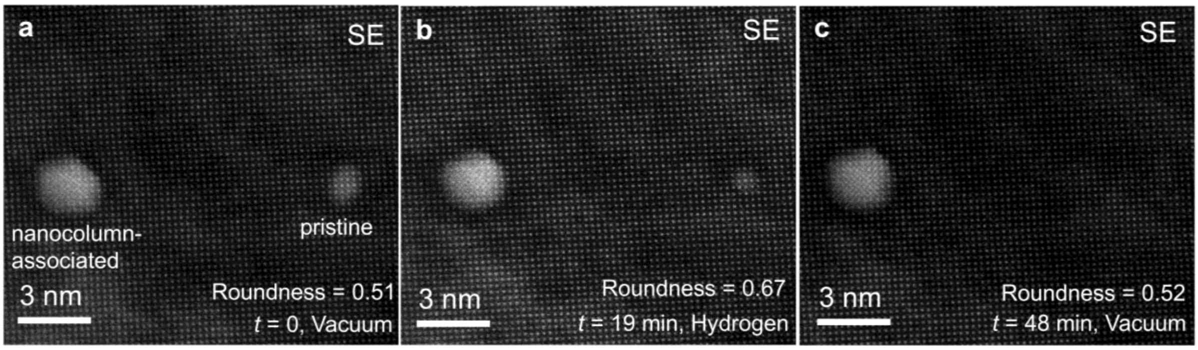

Supplementary Figure 21: The faceting behavior of the particles is observed to change depending on the atmosphere in the microscope. The roundness of the particle (using a comparison between perimeter and area, where Roundness = 1 indicates a circle) is shown to increase in the presence of hydrogen (b), then decrease again when hydrogen is shut off (c). The area was not live imaged in the time between the three images shown here. In addition to the shape change, the pristine particle is observed to disappear between (b) and (c). The temperature was held constant at 500 °C.

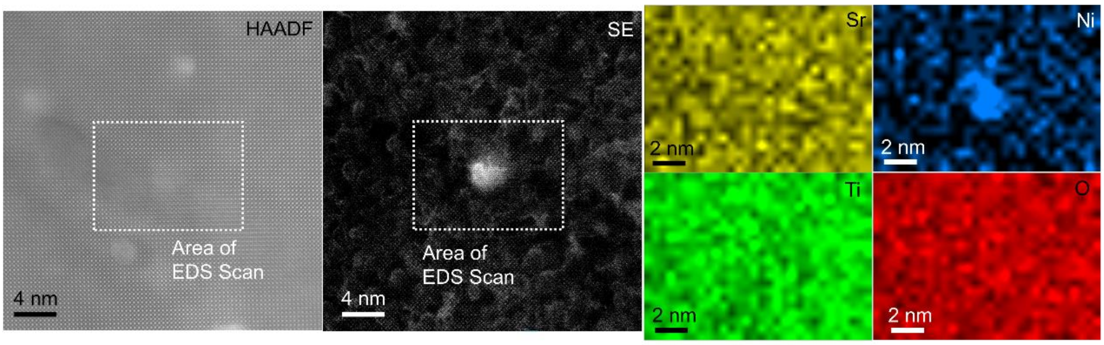

Supplementary Figure 22: HAADF and SE imaging, along with EDS mapping of a pristine exsolved particle in a plan-view orientation. EDS mapping was done at 400 °C in vacuum.

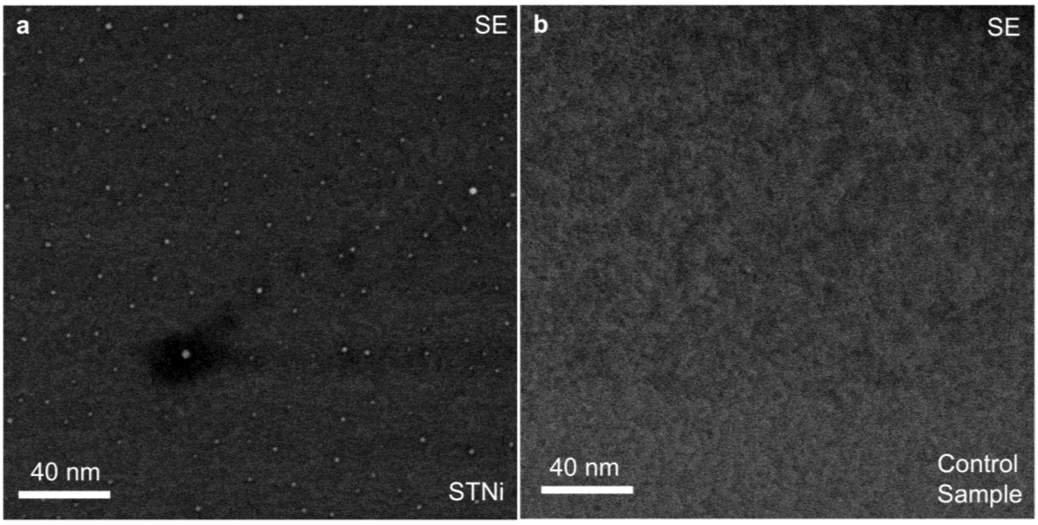

Supplementary Figure 23: A comparison of SE images post-reduction between (a) the STNi sample and (b) a liftout from the Nb:STO substrate. The presence of surface particles only in the case of the STNi film is further evidence of Ni particle formation, and is confirmation that surface particle formation is not an artefact of the sample preparation process. Both images were collected at 600 °C in vacuum.

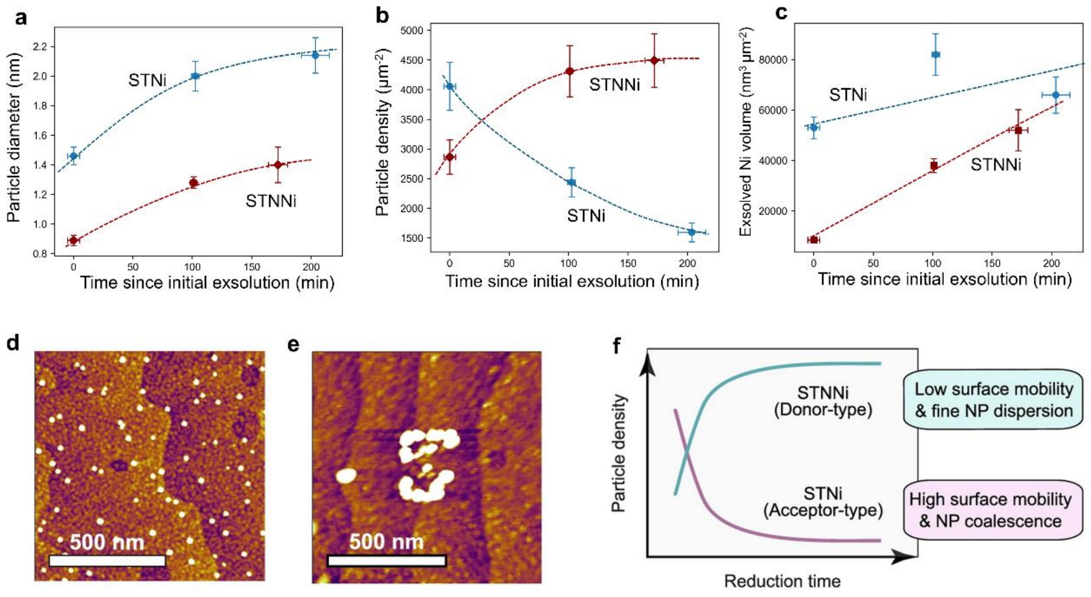

Supplementary Figure 24: Comparison between in-situ and ex-situ results for two material systems. (a-c) comparison of particle size, density, and exsolved volume for a Ni-doped strontium titanate (STNi, the material of focus in this work) and for a Ni/Nb co-doped system (STNNi, the same material with 5% Nb doping). Atomic force microscopy (AFM) images of the STNNi (d) and STNi (e) show evidence of particle migration only in the STNi sample, consistent with in-situ results. Particle density trends in ex-situ experiments (f) match nicely with what is observed during in-situ measurements (b). The images in (d-f) are reprinted from Weber et al.[11] An average of 46 particles per timestep were evaluated for the STNi data, and an average of 92 particles per timestep were evaluated for the STNNi data. Error bars represent the standard error of the mean of the measurement.

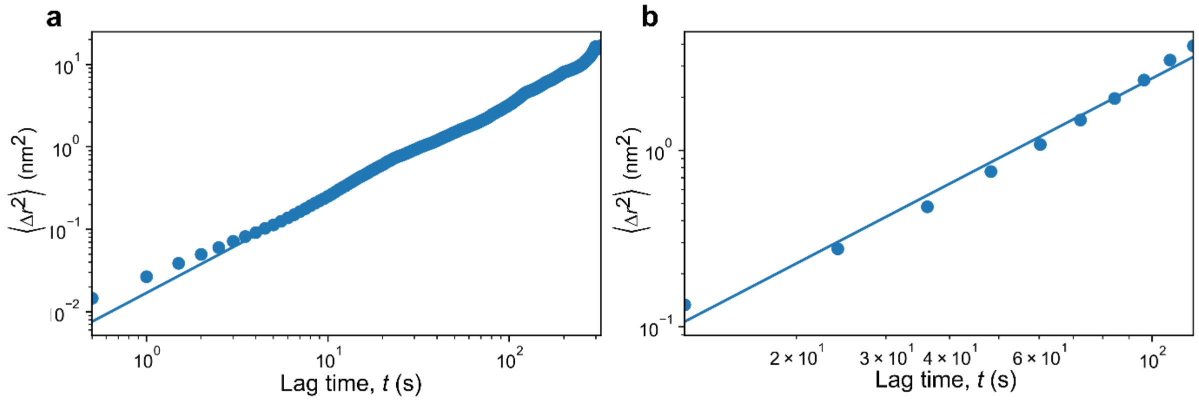

Supplementary Figure 25: Power law fits for random walk kinetics for particle in Fig. 3(a-c) (a) and the assembly of particles in Fig. 3f,g (b). Both groups of particles fit well to a power law, corresponding to random walk kinetics for particle motion.

Supplementary References:

1. Muller, D. A., Nakagawa, N., Ohtomo, A., Grazul, J. L. & Hwang, H. Y. Atomic-scale imaging of nanoengineered oxygen vacancy profiles in SrTiO3. *Nature* **430**, 657–661 (2004).
2. Mogilevsky, P. On the miscibility gap in monazite–xenotime systems. *Phys. Chem. Miner.* **34**, 201–214 (2007).
3. Shannon, R. D. & Prewitt, C. D. Effective ionic radii in oxides and fluorides. *Acta Crystallagr. Sect. B* **25**, (1969).
4. Kowalski, P. M. & Li, Y. Relationship between the thermodynamic excess properties of mixing and the elastic moduli in the monazite-type ceramics. *J. Eur. Ceram. Soc.* **36**, 2093–2096 (2016).
5. He, Z.-D., Tesch, R., Eslamibidgoli, M. J., Eikerling, M. H. & Kowalski, P. M. Low-spin state of Fe in Fe-doped NiOOH electrocatalysts. *Nat. Commun.* **14**, 3498 (2023).
6. Ji, Y. *et al.* Rare-Earth Orthophosphates From Atomistic Simulations. *Front. Chem.* **7**, 197 (2019).
7. Zinkevich, M. Constitution of the Sr–Ni–O system. *J. Solid State Chem.* **178**, 2818–2824 (2005).
8. Takeda, Y., Kanamura, F., Shimada, M. & Koizumi, M. The crystal structure of $BaNiO_3$. *Acta Crystallogr. Sect. B* (1976).
9. Chase, M. W. NIST-JANAF Thermochemical Tables 4th ed. *J Phys. Chem. Reffernce Data* 1529–1564 (1998).
10. Ji, Y., Marks, N. A., Bosbach, D. & Kowalski, P. M. Elastic and thermal parameters of lanthanide-orthophosphate ($LnPO_4$) ceramics from atomistic simulations. *J. Eur. Ceram. Soc.* **39**, 4264–4274 (2019).
11. Weber, M. L. *et al.* Thermal stability and coalescence dynamics of exsolved metal nanoparticles at charged perovskite surfaces. *Nat. Commun.* (2024) doi:10.1038/s41467-024-54008-4.
12. Weber, M. L. *et al.* Reversibility limitations of metal exsolution reactions in niobium and nickel co-doped strontium titanate. *J. Mater. Chem. A* **11**, 17718–17727 (2023).
13. Rheinheimer, W. *et al.* The equilibrium crystal shape of strontium titanate and its relationship to the grain boundary plane distribution. *Acta Mater.* **82**, 32–40 (2015).
14. Jiang, N. Electron beam damage in oxides: a review. *Rep. Prog. Phys.* **79**, 016501 (2016).
15. Hu, S. & Li, W.-X. Sabatier principle of metal-support interaction for design of ultrastable metal nanocatalysts. *Science* **374**, 1360–1365 (2021).
