# Supplementary material for: Direct atomic-scale investigation of the coarsening mechanisms of exsolved catalytic Ni nanoparticles
Source: Nat Commun. 2025 Jul 24;16:6830. doi: 10.1038/s41467-025-61971-z (PMC12290124; doi:10.1038/s41467-025-61971-z)
Supplement: Supplementary file 2 — Description of Additional Supplementary Files [file 41467_2025_61971_MOESM2_ESM.pdf]

Description of Additional Supplementary Files

File Name: Supplementary Movie 1

Description: Unfiltered video of particle movement, with both the HAADF (left) and SE (right) images shown. Two nanocolumn-associated particles are present, and one pristine particle is visible. Note the movement of the pristine particle, and the stability of the two nanocolumn-associated particles. The focus condition was not changed during the video acquisition, so slight drift in image focus is noticeable over the course of the video. Experimental conditions are as follows: *Speed* = 5x, T = 500 °C, Vacuum.

File Name: Supplementary Movie 2

Description: Shortened video clip taken from S1, temporally averaged by two frames and with a gaussian blur applied to improve signal:noise. The video was automatically thresholded using a local Otsu algorithm to identify and outline the mobile particle to emphasize the shape changes during particle movement. Experimental conditions are as follows: *Speed* = 5x, T = 500 °C, Vacuum.

File Name: Supplementary Movie 3

Description: Video demonstrating both particle movement and Ostwald ripening. Both HAADF (left) and SE (right) images are shown, and no filter is applied. The three largest particles grow by OR, the other particles redissolve over the course of the video. One small pristine particle migrates around before redissolving (indicated by the white arrow). Experimental conditions as follows: *Speed* = 20x, T = 400 °C, Hydrogen.

File Name: Supplementary Movie 4

Description: Dynamic change from Fig. 4(d) to Fig. 4(e), showing the partial dissolution of a nanocolumn-associated particle. Experimental conditions as follows: *Speed* = 20x, T = 600 °C, Hydrogen.

File Name: Supplementary Movie 5

Description: Dynamic change starting from Fig. 4(e) showing movement and redissolution of the pristine particle. Experimental conditions as follows: *Speed* = 20x, T = 600 °C, Vacuum.

File Name: Supplementary Movie 6

Description: Overview video containing 49 pristine Ni particles and 2 nanocolumn-associated particles, from which the data in Fig. 3(f-h) was collected. 10 of the 49 pristine particles migrate noticeably over the course of the video. Experimental conditions: *Speed* = 60x, T = 700 °C, Vacuum.

File Name: Supplementary Movie 7

Description: Video of particle ripening, consistent with a nucleation-limited Ostwald ripening behavior. Note the 'bulging' out of the left side of the large particle during the video. Experimental conditions: *Speed* = 30x, T = 600 °C, Vacuum.
